# Molecular evaluation of aminoglycosides resistance and biofilm formation in *Klebsiella pneumoniae* clinical isolates: A cross‐sectional study

**DOI:** 10.1002/hsr2.1266

**Published:** 2023-05-17

**Authors:** Saeed Khoshnood, Sousan Akrami, Morteza Saki, Moloudsadat Motahar, Sara Masihzadeh, Sara Daneshfar, Hossein Meghdadi, Effat Abbasi Montazeri, Marjan Abdi, Zahra Farshadzadeh

**Affiliations:** ^1^ Clinical Microbiology Research Center Ilam University of Medical Sciences Ilam Iran; ^2^ Department of Microbiology, Faculty of Medicine Ahvaz Jundishapur University of Medical Sciences Ahvaz Iran; ^3^ Students' Scientific Research Center (SSRC) Tehran University of Medical Sciences Tehran Iran; ^4^ Infectious and Tropical Diseases Research Center, Health Research Institute Ahvaz Jundishapur University of Medical Sciences Ahvaz Iran

**Keywords:** aminoglycoside modifying enzymes, aminoglycoside resistance, biofilm, *Klebsiella pneumoniae*, 16S rRNA methylases

## Abstract

**Background and Aims:**

Resistance to antibiotics and the capability to develop biofilm as two main virulent determinants of *Klebsiella pneumoniae* have important role in infection persistence. The aim of the study was to evaluate the association between the prevalence of aminoglycoside resistance and virulence genes and biofilm formation capacity in *K. pneumoniae* strains isolated from hospitalized patients in South‐West of Iran.

**Methods:**

A total of 114 non‐duplicate clinical isolates of *K. pneumoniae* collected from Ahvaz teaching hospitals. Identification of species was performed by biochemical tests and then confirmed by polymerase chain reaction (PCR) of *rpoB* gene. The susceptibility to antibiotics was determined by Kirby−Bauer disk diffusion method. Biofilm formation was assessed by microtiter plate method. Finally, PCR was conducted to detect virulence gene determinants including fimbrial genes, aminoglycoside modifying enzymes‐ and 16S rRNA methylase (RMTase) genes.

**Results:**

Totally, all collected strains were carbapenem resistant and showed multidrug‐ and extensively drug‐resistance phenotype (75% and 25%, respectively). Seventy‐one percent (*n* = 81) of isolates were non‐susceptible to aminoglycosides. Among aminoglycoside antibiotics, *K. pneumoniae* isolates showed the highest and lowest resistance rates to tobramycin (71%) and the amikacin (25%), respectively. All biofilm producer strains were positive for the presence virulence determinants including *ecpA, fimA, mrkD*, and *mrkA*. Of 81 aminoglycosides non‐susceptible isolates 33% were positive for the presence *ant (2″)‐Ia* as the most prevalent gene followed by *aac (3′)‐IIa* and *armA* (27%), *aac (6′)‐Ib* (18%), and *aph (3′)‐Ia* (15%).

**Conclusion:**

*K. pneumoniae* isolates showed the highest and the lowest aminoglycoside resistance rates to tobramycin and amikacin, respectively. Majority of isolates were biofilm producers and there was significant association between antibiotic resistance pattern and the strength of biofilm production. The *ant(2″)‐Ia, aac (3′)‐IIa*, and *armA* genes in aminoglycoside‐resistant isolates.

## 1 INTRODUCTION

*Klebsiella pneumoniae* is an opportunistic human pathogen responsible for approximately one‐third of nosocomial infections such as pneumonia, urinary tract, bloodstream, and wound infections.^1^

Nosocomial *K. pneumoniae* is cause of one‐third of gram‐negative infections and is related to carbapenem resistance global.^2^ The gastrointestinal tract of patients and the hands of medical personnel are the primary reservoirs for *K. pneumoniae* transmission. This microorganism is especially dangerous in immunocompromised patients and those who have indwelling medical devices on which biofilm could developed.^3^, ^4^ The occurrence and increase in multidrug‐resistant (MDR) bacteria have become a global public health issues. The development of antimicrobial resistance in *K. pneumoniae* isolates poses a serious problem to public health, mainly with the rise of carbapenem‐resistant *K. pneumoniae* (CRKP) isolates. The irrational use of antibiotics in clinical practice has fostered the spread of resistance to “old class antibacterials.”^5^

Clonal complex 258 (CC258) is one of the CRKP clones disseminating all over the world.^6^ In particular, CRKP exhibiting resistance to colistin and tigecycline have raised important clinical concern, as these are the last‐line drugs for the treatment of these isolates.^7^ The worldwide prevalence of colistin‐resistant *K. pneumoniae* simplified by chromosomal and plasmid‐mediated LPS changes have steadily increased with increased colistin usage for treating CRKP isolates.^8^ Colistin‐resistant *K. pneumoniae* infections are increasing in intensive care units, particularly in patients who ultimately depend on vascular catheters for long‐term or short‐term use.^9^

CRKP isolates can cause serious infections in association with prolonged hospital stays, narrow therapeutic choices, and increased mortality. Several investigations indicated that MDR bacteria isolated from hosts with persistent infections are often biofilm formers. Also, it was noted that intrinsic resistance to antibacterial drugs increases when *K. pneumoniae* isolates grow as biofilms.^10^ Biofilm enable the *K. pneumonia* isolates to survive in the rough environmental conditions such as the antibiotic pressure and the host immune response. Biofilm formation in therapeutic devices, including urinary catheters, as their important stage in their disease pathogenesis can result in biofilm‐associated drug tolerance in these isolates.^11^ In *K. pneumoniae*, biofilm development is an important virulence characteristic, and managing biofilms is very challenging as they confer significant tolerance to drug effects and host defense responses.^8^

Biofilm can be considered as a reservoir of genetic diversity. In biofilm, the emergence and spread of antibiotic resistance genes increase through horizontal gene transfer. The horizontal transmission of antibiotic resistance genes increase between bacterial cells in an extracellular polymeric substance matrix.^12^

In addition to biofilm, the ability to acquire antibiotics resistance is considered as another important virulence determinant which plays a significant role in the successful pathogenicity. Aminoglycosides are frequently used to treat infections caused by Enterobacteriaceae.^13^ These antibiotics are one of the important groups of the broad‐spectrum antibiotics inhibiting protein synthesis by binding to the 30S ribosomal subunit in a wide range of gram‐negative bacteria, especially Enterobacteriaceae. However, the improper use of them could lead to the emergence of resistant strains, leading to limitation in the choice of treatment options for life threating infectious diseases. Acquired resistance to aminoglycosides has been reported in these bacteria. The three mechanisms of resistance include the reduction of antibiotic affinity in binding to the ribosomal site, 16S rRNA, reduction in antibiotic permeability, and enzymatic inactivation of the antibiotic are responsible for resistance to aminoglycosides.^14^

Enzymatic inactivation of aminoglycosides through three main known classes of aminoglycosides modifying enzymes (AMEs) including N‐acetyltransferases (AACs), O‐nucleotidyltransferases (ANTs), and O‐phosphotransferases (APHs) is the most considerable mechanism of bacterial resistance to aminoglycosides. AACs cause resistance to aminoglycosides by transferring acetyl groups from acetyl‐coenzyme A to amino group of these antibiotics, while ANTs by transferring adenyl group from ATP, and APHs by transferring γ‐phosphate to hydroxyl groups of these antibiotics confer the resistance, respectively.^15^, ^16^, ^17^

Another recently emerged mechanism of aminoglycosides resistance is methylation of ribosomal binding site of the antibiotic by 16S RMTase. The six important genes encoding 16S RMTase including *armA, rtmA, rtmB, rtmC* and *rtmD*, and *npmA*, are located on transposons within transferable plasmids which render high level of resistance to all clinically relevant aminoglycosides.^14^ Today, there is little updated information about the prevalence of the gene encoding AMEs and 16S RMTase in *K. pneumoniae* from Iran. The objectives of this study were to: (1) determination of antibiotic susceptibility pattern and capacity to form biofilm, (2) detection of AME and virulence genes, (3) identification of the relationship between the biofilm formation and aminoglycoside resistance among *K. pneumoniae* strains isolated from hospitalized patients in Iran.

## 2 MATERIALS AND METHODS

### 2.1 Bacterial strains

This descriptive cross‐sectional study was performed on clinical isolates of *K. pneumoniae* collected from different clinical specimens in Ahvaz teaching hospitals affiliated to Ahvaz Jundishapur University of Medical Sciences during 6 months between October 2020 and March 2021. This project confirmed by the Ethics Committee of Ahvaz Jundishapur University of Medical Sciences, Ahvaz, Iran (No. IR.AJUMS.REC.1398.724). Patients signed informed consent forms before the start of this research project. The research was carried out in accordance with the Helsinki Declaration. Among all collected samples, 114 isolates were identified as *K. pneumoniae* using by phenotypic methods.

### 2.2 Antimicrobial susceptibility testing

The antibiotic susceptibility test was done by the disk diffusion method on the Mueller−Hinton agar plates (Merck) according to CLSI guideline^18^ for amikacin (AMK: 30 μg), gentamycin (GEN: 10 μg), tobramycin (TOB: 10 μg), piperacillin‐tazobactam (PTZ:110), imipenem (IMI: 10 μg), meropenem (MER: 10 μg), ertapenem (ERT: 10 μg), cefazolin (CZ:30 μg), cefoxitin (FOX: 30 μg), cefotaxime (CTX:30 μg), ceftazidime (CAZ: 30 μg), ceftriaxone (CRO: 30 μg), cefoperazone (CFP: 75 μg), ciprofloxacin (CIP: 5 μg), azithromycin (AZ: 15 μg), ampicillin‐sulbactam (AMP/SL: 20 μg), and tigecycline (TGC: 15 μg) (MAST Diagnostics). *Escherichia coli* ATCC 25922 was used as a quality control strain.

### 2.3 Quantification of the biofilm mass

The capacity of isolates to develop biofilm was measured using the microtiter plate (MTP) method. The isolates were classified into four categories using the previously described criteria by Haney et al.^19^ as follows: no biofilm producers (A ≤ Ac), weak biofilm producers (Ac < A ≤ 2Ac), moderate biofilm producers (2Ac < A ≤ 4Ac), and strong biofilm producers (A > 4Ac). The PAO1 strain of *Pseudomonas aeruginosa* was also used as a positive control for the biofilm assay. All samples were tested three times.

### 2.4 Biofilm eradication assay

The MTP method was used to evaluate the capacity of amikacin and gentamicin to eradicate biofilms, as described previously.^20^ Following biofilm formation for 48 h, the medium was discarded, and the wells were washed with PBS to remove nonattached bacteria. Then, the plates were filled with 100 μL of MHB in the presence of 2% glucose and 100 μL of antibiotics (0.25−512 μg/mL). After incubation at 37°C for 24 h, the plates were treated for the biofilm formation assay as described above. The minimal biofilm eradication concentration (MBEC) was defined as the lowest concentration of antibiotics leading to a decrease in the metabolic activity of preformed biofilm by 100%. The experiment was done in triplicate and repeated three times.

### 2.5 Detection of biofilm specific, and aminoglycoside resistance determinants

The polymerase chain reaction (PCR) was performed for detection of genes involved in biofilm formation (*fimA, fimH, mrkA, mrkD*, and *ecpA*) in all isolates, AME genes [*aac (3′)‐IIa, ant(2″)‐Ia, aph(3′)‐Ia, aac(6′)‐Ib*] and 16S rRNA methylase genes (*npmA, armA, rmtA, rmtB, rmtC, rmtD*) in aminoglycosides resistant isolates, in a final volume of 25 µL, including the following ingredients: 12.5 μL of 2 × Master Mix (Amplicon), 1 μL of forward primer, 1 μL of reverse primer, 2 µL of DNA template, and 8.5 μL sterile distilled water. The following thermal program was then used to amplify the fragments: Initial denaturation temperature of 95°C for 5 min, followed by denaturation temperature of 95°C for 45 s, annealing temperature of 56−63.5°C for 30 s, elongation temperature of 72°C for 45 s, and final elongation temperature of 72°C for 5 min. The number of cycles selected to perform the reaction was 35 cycles. The oligonucleotide primer sequences used in the present work are shown in Table 1.^21^, ^22^, ^23^, ^24^ Sequencing of both sense and antisense strands of purified PCR product was performed to verify detected genes (Macrogen Company). Sequence confirmation was followed by alignment and analysis using the BLAST program of the National Center for Biotechnology Information (http://blast.ncbi.nlm.nih.gov/Blast).

**Table 1:** Sequence of primers used in this study.

Gene	Primer sequence (5′‐3′)	Product size (bp)	References
*ecpA*	F‐GCAACAGCCAAAAAAGACACC	477	[21]
	R‐CCAGGTCGCGTCGAACTG		
*mrkA*	F‐CGGTAAAGTTACCGACGTATCTTGTACTG	498	[21]
	R‐GCTGTTAACCACACCGGTGGTAAC		
*mrkD*	F‐CTGACGCTTTTTATTGGCTTAATGGCGC	757	[21]
	R‐GCAGAATTTCCGGTCTTTTCGTTTAGTAG		
*fimA*	F‐CGGACGGTACGCTGTATTTT	500	[21]
	R‐GCTTCGGCGTTGTCTTTATC		
*fimH*	F‐CGCCTGGTCCTTTGCCTGCA	817	[22]
	R‐CTGCACGTTGCCGGCGGTAA		
*aac(3′)‐IIa*	F‐ATGCATACGCGGAAGGC	822	[23]
	R‐TGCTGGCACGATCGGAG		
*ant(2″)‐Ia*	F‐ATCTGCCGCTCTGGAT	404	[23]
	R‐CGAGCCTGTAGGACT		
*aph(3′)‐Ia*	F‐CGAGCATCAAATGAAACTGC	623	[23]
	R‐GCGTTGCCAATGATGTTACAG		
*aac(6′)‐Ib*	F‐TATGAGTGGCTAAATCGAT	395	[23]
	R‐CCCGCTTTCTCGTAGCA		
*npmA*	F‐GGAGGGCTATCTAATGTGGT	371	[24]
	R‐GCCCAAAGAGAATTAAACTG		
*armA*	F‐AGGTTGTTTCCATTTCTGAG	591	[24]
	R‐TCTCTTCCATTCCCTTCTCC		
*rmtA*	F‐CTAGCGTCCATCCTTTCCTC	635	[24]
	R‐TTTGCTTCCATGCCCTTGCC		
*rmtB*	F‐CCCAAACAGACCGTAGAGGC	585	[24]
	R‐CTCAAACTCGGCGGGCAAGC		
*rmtC*	F‐CGAAGAAGTAACAGCCAAAG	711	[24]
	R‐ATCCCAACATCTCTCCCACT		
*rmtD*	F‐CGGCACGCGATTGGGAAGC	401	[24]
	R‐CGGAAACGATGCGACGAT		

### 2.6 Statistical analysis

Descriptive data were analyzed using SPSS version 22.0 statistics software (IBM Corporation). The results were presented as descriptive statistics in terms of relative frequency (mean ± standard deviation, percentile). Fisher's exact test was employed to analyze the significant differences between variables. In addition, *p*‐value < 0.05 was considered as significance level.

## 3 RESULTS

### 3.1 Bacterial characterization

Between October 2020 and March 2021, a total of 114 clinical isolates of *K. pneumoniae* were obtained from several clinical specimens which distribution of them in terms of sample type shown in Table 2. These isolates were recovered from 59 (52%) male and 55 (4%) female hospitalized patients which their mean age was 52 years (range, 10−89 years, mean ± SD: 49.09 ± 22.76). *K. pneumonia* mostly were isolated from urine (36.8%, *n* = 42/114), sputum (22.8%, *n* = 26/114), and tracheal tube (21.1%, *n* = 24/114). Remaining specimens were collected from blood (10.5%, *n* = 12/114), peritoneal aspirate (4.4%, *n* = 5/114), wound (1.8%, *n* = 2/114), tonsil (1.8%, *n* = 2/114), and surgical drain (0.9%, *n* = 1/114). The sputum and tracheal tube samples were obtained from patients with age mean ± SD: 61.07 ± 8.25 and 66 ± 7.88 years old, respectively.

**Table 2:** Distribution of samples in terms of gender.

Sample	Gender	
	Male *N* (%)	Female *N* (%)
Urine	9 (37.5)	15 (62.5)
Blood	9 (75)	3 (25)
Peritoneal aspirate	2 (40)	3 (60)
Sputum	37 (88)	5 (12)
Tracheal tube	25 (96.2)	1 (3.8)
Wound	1 (50)	1 (50)
Tonsil	2 (100)	0 (0.0)
Surgical drain	1 (100)	0 (0.0)

### 3.2 Antimicrobial susceptibility testing

*K. pneumoniae* strains in present study were resistant to at least one of the carbapenem antibiotic. So, all of our examined strains were categorized as carbapenem resistant. However, according to the International Expert proposal for Interim Standards Guidelines,^25^ 75.4% (*n* = 86/114) and 24.6% (*n* = 28/114) of isolates revealed MDR and XDR phenotypes, respectively. In total, 74.6% (85/114) of isolates were resistant to aminoglycosides. Among aminoglycoside antibiotics, *K. pneumoniae* isolates showed the highest and the lowest resistance rates to tobramycin (71.1%, *n* = 81/114) and the amikacin (24.6%, *n* = 28/114), respectively. The *K. pneumoniae* isolates showed different levels of resistance to antibiotics as follows: gentamycin (36.8%, *n* = 42/114), tigecycline (33.3%, *n* = 38/114), piperacillin‐tazobactam (51.8%, *n* = 59/114), imipenem (68.4%, *n* = 78/114), meropenem (100.0%, *n* = 114/114), ertapenem (55.3%, *n* = 63/114), cefazolin (55.3%, *n* = 63/114), cefoxitin (57.0%, *n* = 65/114), cefotaxime (75.4%, *n* = 86/114), ceftazidime (78.1%, *n* = 89/114), ceftriaxone (79.8%, *n* = 91/114), cefoperazone (86.0%, *n* = 98/114), ciprofloxacin (41.2%, *n* = 47/114), azithromycin (20.2%, *n* = 23/114), and ampicillin‐sulbactam (78.9%, *n* = 90/114).

### 3.3 Biofilm formation assay

According to our results, 77.2% (*n* = 88/114) of isolates were biofilm producer. Of these, 65.9% (*n* = 58/88) of isolates were strongly able to produce biofilm and remaining isolates were considered as moderate (27.3%, *n* = 24/88) and weak (6.8%, *n* = 6/88) biofilm formers. There was significant relationship between resistance phenotype and different degrees of biofilm production, so that the prevalence of strong biofilm producers among extensively drug resistant (XDR) phenotype was higher than MDR group (82%, *n* = 23/28 in XDR vs. 40%, *n* = 34/86 in MDR, *p* = 0.0001). Majority of non‐biofilm producer isolates (88.5%, *n* = 23/26) were exhibited MDR phenotype. Biofilm producer strains were significantly placed in aminoglycosides non‐susceptible isolates (*p* < 0.001; the data are shown in Table 3). It is noteworthy that only 11.5% (*n* = 3/26) of non‐biofilm producer isolates were resistant to aminoglycosides.

**Table 3:** Distribution of aminoglycoside susceptibility based on biofilm formation capacity.

Biofilm	**Tobramicin (*n*)**		**Amikacin (*n*)**		**Gentamicin (*n*)**	
	N	S	N	S	N	S
Producer	74	14	28	60	42	46
Non‐producer	8	18	0	26	0	26
*p* Value	<0.001		<0.001		<0.001	

Abbreviations: N, non‐susceptible; S, susceptible.

### 3.4 Ability of amikacin and gentamicin for biofilm eradication

To investigate the ability of gentamicin and amikacin in eradication of preformed biofilm, we determined MBEC. So those, after treatment with antibiotics at the different concentrations of amikacin and gentamicin, living bacteria were counted in all tested isolates. Table 5 shows the capacity of mentioned antibiotics to disperse preformed biofilm in *K. pneumonia* isolates. Gentamicin eradicated the preformed biofilm with MBEC values between 0.25 and 32 μg/mL (MBEC_50_ = 8 and MBEC_90_ = 32) and for amikacin between 0.25 and 128 μg/mL (MBEC_50_: 16 and MBEC_90_: 64). Data indicated that antibiotics‐resistant isolates in the planktonic mode did not show increase in resistance to antibiotics when grow in biofilm state. However, stronger biofilm former showed a dramatic increase in resistance to antibiotics when grow in biofilms mode compared with moderate and weak biofilm former strains. The gentamicin and amikacin MBECs against stronger biofilm former strains were ≥16 and ≥32 μg/mL, respectively, while, against moderate and weak biofilm former, the gentamicin MBEC was ≤8 μg/mL and the amikacin MBEC was ≤16 μg/mL.

### 3.5 Distribution of AMEs and biofilm related genes in K. pneumonia

The results of our study showed that among 85 isolates that were resistant to at least one of the aminoglycoside antibiotics, 82.4% (*n* = 70/85) had AME genes. Some isolates had only one gene and some others harbored a combination of two or more genes (Table 4). The presence of these genes was not confirmed in remaining aminoglycoside non‐susceptible isolates (*n* = 15/85; 17.6%) by PCR and sequencing. Of the 70 positive isolates for at least one of the AMEs or RMTase genes, only 2 isolates carried one gene encoding AMEs and 15 isolates harbored *armA* alone, 38 isolates carried two genes, and 15 isolates carried three genes. Overall, 10 different combination patterns were determined in mentioned isolates (Table 4).

**Table 4:** The distribution of AMEs and 16S rRNA methylase gene in 85 aminoglycoside‐resistant *Klebsiella pneumoniae* isolates.

AMEs and RMTase genes	*N* (%)
*armA*	15 (17.6)
*armA+ ant(2″)‐Ia*	13 (15.3)
*armA+ aac(6′)‐Ib*	3 (3.5)
*ant(2″)‐Ia + aac(6′)‐Ib*	5 (5.9)
*ant(2″)‐Ia + aac(3′)‐IIa*	6 (7.1)
*ant(2″)‐Ia + aph(3′)‐Ia*	2 (2.4)
*ant(2″)‐Ia + aph(3′)‐Ia + aac(3′)‐IIa*	5 (5.9)
*aac(6′)‐Ib + aac(3′)‐IIa*	9 (10.6)
*aac(6′)‐Ib + aac(3′)‐IIa + aph(3′)‐Ia*	4 (4.7)
*aac(3′)‐IIa + ant(2″)‐Ia + aph(3′)‐Ia*	3 (3.5)
*ant(2″)‐Ia + aac(3′)‐IIa +aph(3′)‐Ia*	3 (3.5)
*ant(2″)‐Ia*	1 (1.2)
*aac(3′)‐IIa*	1 (1.2)

Abbreviation: AME, aminoglycoside modifying enzymes.

The prevalence of genes encoding AMEs among 85 aminoglycoside non‐susceptible *K. pneumoniae* isolates were as follows: *ant(2”)‐Ia* (*n* = 38; 44.7%), *aac(3′)‐IIa* (*n* = 31, 36.5%), *armA* (*n =* 31, 36.5%), *aac(6′)‐Ib* (*n* = 21, 24.7%), and *aph(3′)‐Ia* (*n* = 17; 20%). None of the mentioned isolates were positive for *rmtA, rmtB, rmtC, rmtD*, and *npmA* genes. Our findings revealed that the most frequent AME related genes and RMTase gene were *ant(2”)‐Ia, aac(3′)‐IIa*, and *armA* genes followed by *aac(6′)‐Ib*. As shown in Table 4, *ant(2”)‐Ia* was found to coexist with *aac*(3′)‐IIa (*n* = 6; 7.1%), *aph(3′)‐Ia* (*n* = 2; 2.4%), and with *aac(6′)‐Ib* (*n* = 5, 5.9%). The mentioned coexistences were the most common combinations in tobramycin and gentamicin non‐susceptible isolates. All amikacin resistant isolates harbored *armA* either alone or in combination with *ant*(2”)‐Ia and *aac(6′)‐Ib*. It is noteworthy that, isolates positive for *ArmA* in combination with mentioned AMEs genes were simultaneous resistance to tobramycin and amikacin.

In the current study, the prevalence of genes involved in biofilm formation and virulence (*ecpA, fimA, mrkD*, and *mrkA*) were detected in all biofilm producer *K. pneumoniae* strains, whereas *fimH* was determined in 93.2% (*n* = 82/88) of biofilm forming isolates. *FimA* and *ecpA* were detected in 42.3% (*n* = 11/26) and 38.5% (*n* = 10/26) of non‐biofilm producer strains, respectively. Among these strains *mrkA, fimH*, and *mrkD* genes were not identified. Most isolates harboring AMEs genes were also positive for presence biofilm related genes including *ecpA, mrkA*, and fimbrial genes. Table 5 listed all the strains analyzed, with origin, genotype(s), and phenotype(s).

**Table 5:** Demographic data, prevalence of fimbrial and AME genes, and biofilm production in *Klebsiella pneumoniae* isolates.

Isolate number	Ward	Sample	Patient gender	Age	Biofilm	MBEC		Resistotype	Presence of AME genes
						Gentamicin	Amikacin		
Kp 121	Men	Urine	Male	43	Weak	2	4	MDR	*ecpA*
Kp 122	Women	Sputum	Female	42	Weak	2	8	MDR	*ecpA*
Kp 123	Men	Peritoneal aspirate	Male	36	Moderate	1	8	MDR	*ecpA*
Kp 126	Pediatrics	Urine	Female	13	Moderate	4	16	MDR	*ecpA, ant(2”)‐Ia*
Kp 128	Pediatrics	Sputum	Female	11	Moderate	8	32	MDR	*ecpA, aac(3′)‐IIa*
Kp 129	ICU	Sputum	Female	56	Weak	4	16	MDR	*ecpA*
Kp 130	ICU	Sputum	Male	74	Moderate	8	32	MDR	*ecpA*
Kp 131	ICU	Tracheal tube	Female	82	Weak	4	16	MDR	*ecpA, FimA, ant(2”)‐Ia*
Kp 133	Surgery	Peritoneal aspirate	Female	53	Weak	8	32	MDR	*ecpA, FimA, FimH, ant(2”)‐Ia, aph(3′)‐Ia, aac(6′)‐Ib*
Kp 136	Men	Urine	Male	51	Strong	32	128	XDR	*ecpA, FimA*
Kp 137	Men	Urine	Male	50	Weak	8	8	MDR	*ecpA*
Kp 140	Burn	Wound	Female	21	Strong	64	64	MDR	‐
Kp 141	Burn	Wound	Male	19	Moderate	8	32	MDR	*ecpA, ant(2”)‐Ia*
Kp 142	ENT	Tonsil	Male	10	Non	16	16	MDR	*ecpA, ant(2”)‐Ia*
Kp 143	Men	wound	Male	23	Moderate	4	16	MDR	*ecpA, ant(2”)‐Ia*
Kp 145	ENT	Tonsil	Male	13	Non	4	8	MDR	*ecpA, FimA, FimH, ant(2”)‐Ia, aac(6′)‐Ib*
Kp 146	Men	Urine	Male	29	Moderate	32	8	XDR	*ecpA*
Kp 147	Surgery	peritoneal aspirate	Female	42	Strong	16	128	MDR	*ecpA*
Kp 148	ICU	Sputum	Female	58	Strong	16	8	MDR	*ecpA, aac(3′)‐IIa, ant(2”)‐Ia, aph(3′)‐Ia*
Kp 149	Internal medicine	Urine	Female	34	Moderate	8	16	MDR	*ecpA, FimA, ant(2”)‐Ia*
Kp 150	Men	Sputum	Male	43	Moderate	16	8	XDR	*ecpA, FimA, FimH, aac(3′)‐IIa, aac(6′)‐Ib*
Kp 151	Women	Urine	Female	20	Strong	16	64	MDR	*ecpA, ant(2”)‐Ia, aph(3′)‐Ia*
Kp 152	ICU	Tracheal tube	Male	68	Moderate	16	8	MDR	*ecpA*
Kp 158	Men	Urine	Male	35	Moderate	8	8	MDR	*ecpA, aac(3′)‐IIa, ant(2”)‐Ia*
Kp 160	Internal medicine	Urine	Female	32	Strong	16	32	MDR	*ecpA, FimA, ant(2”)‐Ia, aph(3′)‐Ia*
Kp 161	Women	Urine	Female	41	Moderate	2	4	MDR	*ecpA, aac(3′)‐IIa, ant(2”)‐Ia*
Kp 163	Women	urine	Female	27	Moderate	32	64	MDR	*aac(3′)‐IIa, ant(2”)‐Ia*
Kp 167	Men	Urine	Male	42	Strong	16	64	MDR	*ecpA, aac(3′)‐IIa, ant(2”)‐Ia*
Kp 168	Surgery	Surgical drain	Male	51	Non	4	8	MDR	*ecpA*
Kp 169	Urology	Urine	Female	34	Strong	16	64	MDR	*ecpA, FimA*
Kp 170	Urology	Urine	Female	22	Strong	8	8	MDR	*ecpA, FimA, FimH, aac(3′)‐IIa, ant(2”)‐Ia, aac(6′)‐Ib*
Kp 172	Urology	Urine	Female	25	Strong	8	32	MDR	*ecpA, FimA, ant(2”)‐Ia, aph(3′)‐Ia*
Kp 180	ICU	Sputum	Male	81	Strong	8	16	MDR	*ecpA, FimA, FimH, aph(3′)‐Ia, aac(6′)‐Ib*
Kp 181	Men	Sputum	Male	40	Strong	4	32	MDR	*ecpA*
Kp 182	URology	Urine	Female	31	Moderate	4	32	MDR	*ecpA, aac(3′)‐IIa*
Kp 1	Internal medicine	Urine	Male	45	Moderate	4	16	MDR	‐
Kp2	Internal medicine	Sputum	Male	26	Moderate	4	32	MDR	‐
Kp3	ICU	Tracheal tube	Male	52	Non	4	2	MDR	‐
Kp 4	Men	Urine	Male	41	Moderate	8	16	MDR	‐
Kp 125	Women	Peritoneal aspirate	Male	37	Strong	16	16	MDR	‐
Kp 132	Urology	Urine	Male	67	Moderate	32	4	MDR	‐
Kp 134	Surgery	Peritoneal aspirate	Female	38	Moderate	2	8	MDR	‐
Kp 135	Internal medicine	Sputum	Male	35	Strong	16	64	MDR	‐
Kp 138	Infectious diseases	Urine	Female	41	Moderate	4	16	MDR	‐
Kp 139	Infectious diseases	Urine	Female	29	Strong	16	32	MDR	‐
Kp 155	Urology	Urine	Female	38	Strong	32	128	MDR	‐
Kp 159	Men	Urine	Female	42	Strong	16	64	MDR	‐
Kp 164	Women	Sputum	Female	50	Moderate	8	32	MDR	‐
Kp 171	Men	Sputum	Male	48	Strong	64	128	MDR	‐
Kp 178	Internal medicine	Urine	Female	38	Strong	16	32	MDR	‐
Kp 201	Men	Urine	Male	43	Strong	16	32	MDR	*ecpA*
Kp 202	Women	Sputum	Female	42	Strong	4	64	MDR	*ecpA*
Kp 203	Men	Sputum	Male	36	Moderate	16	32	MDR	*ecpA*
Kp 204	Pediatrics	Urine	Female	13	Moderate	2	4	MDR	*ecpA, ant(2”)‐Ia*
Kp 205	Pediatrics	Blood	Female	11	Moderate	4	8	MDR	*ecpA, aac(3′)‐IIa*
Kp 206	ICU	Sputum	Female	56	Strong	4	8	MDR	*ecpA*
Kp 207	ICU	Sputum	Male	74	Moderate	8	8	MDR	*ecpA*
Kp 208	ICU	Tracheal tube	Female	82	Strong	32	64	MDR	*ecpA, FimA, ant(2”)‐Ia*
Kp 209	Surgery	Peritoneal aspirate	Female	53	Strong	16	8	MDR	*ecpA, FimA, FimH, ant(2”)‐Ia, aph(3′)‐Ia, aac(6′)‐Ib*
Kp 210	Men	Urine	Male	51	Strong	16	32	XDR	*ecpA, FimA*
Kp 211	Men	Urine	Male	50	Strong	32	128	MDR	*ecpA*
Kp 212	Burn	Wound	Female	21	Strong	16	2	MDR	‐
Kp 213	Burn	Wound	Male	19	Moderate	32	64	MDR	*ecpA, ant(2”)‐Ia*
Kp 214	ENT	Tonsil	Male	10	Non	4	2	MDR	*ecpA, ant(2”)‐Ia*
Kp 215	Men	Blood	Male	23	Moderate	16	32	MDR	*ecpA, ant(2”)‐Ia*
Kp 216	ENT	Tonsil	Male	13	Non	4	4	MDR	*ecpA, FimA, FimH, ant(2”)‐Ia, aac(6′)‐Ib*
Kp 217	Men	Urine	Male	29	Moderate	8	4	XDR	*ecpA*
Kp 218	Surgery	Sputum	Female	42	Strong	8	32	MDR	*ecpA*
Kp 219	ICU	Sputum	Female	58	Non	4	64	MDR	*ecpA, aac(3′)‐IIa, ant(2”)‐Ia, aph(3′)‐Ia*
Kp 220	Internal medicine	Urine	Female	34	Moderate	16	32	MDR	*ecpA, FimA, ant(2”)‐Ia*
Kp 221	Men	Blood	Male	43	Moderate	4	8	XDR	*ecpA, FimA, FimH, aac(3′)‐IIa, aac(6′)‐Ib*
Kp 222	Women	Urine	Female	20	Strong	8	64	MDR	*ecpA, ant(2”)‐Ia, aph(3′)‐Ia*
Kp 223	ICU	Tracheal tube	Male	68	Moderate	4	64	MDR	*ecpA*
Kp 224	Men	Urine	Male	35	Moderate	8	16	MDR	*ecpA, aac(3′)‐IIa, ant(2”)‐Ia*
Kp 225	Internal medicine	Urine	Female	32	Strong	16	32	MDR	*ecpA, FimA, ant(2”)‐Ia, aph(3′)‐Ia*
Kp 226	Women	Urine	Female	41	Moderate	2	4	MDR	*ecpA, aac(3′)‐IIa, ant(2”)‐Ia*
Kp 227	Women	urine	Female	27	Moderate	4	8	MDR	*aac(3′)‐IIa, ant(2”)‐Ia*
Kp 228	Men	Urine	Male	42	Non	4	32	MDR	*ecpA, aac(3′)‐IIa, ant(2”)‐Ia*
Kp 229	Surgery	Tonsil	Male	51	Non	1	4	MDR	*ecpA*
Kp 230	Urology	Urine	Female	34	Non	4	8	MDR	*ecpA, FimA*
Kp 231	Urology	Urine	Female	22	Strong	8	16	MDR	*ecpA, FimA, FimH, aac(3′)‐IIa, ant(2”)‐Ia, aac(6′)‐Ib*
Kp 232	Urology	Urine	Female	25	Strong	16	64	MDR	*ecpA, FimA, ant(2”)‐Ia, aph(3′)‐Ia*
Kp 233	ICU	Sputum	Male	81	Strong	32	32	MDR	*ecpA, FimA, FimH, aph(3′)‐Ia, aac(6′)‐Ib*
Kp 234	Men	Blood	Male	40	Non	4	8	MDR	*ecpA*
Kp 235	Urology	Urine	Female	31	Moderate	64	16	MDR	*ecpA, aac(3′)‐IIa*
Kp 236	Internal medicine	Urine	Male	45	Moderate	8	32	MDR	‐
Kp237	Internal medicine	Blood	Male	26	Moderate	16	32	MDR	‐
Kp238	ICU	Tracheal tube	Male	52	Non	4	16	MDR	‐
Kp 239	Men	Urine	Male	41	Moderate	16	64	MDR	‐
Kp 240	Women	Sputum	Male	37	Strong	64	32	MDR	‐
Kp 241	Urology	Urine	Male	67	Moderate	16	32	MDR	‐
Kp 242	Surgery	Sputum	Female	38	Moderate	16	64	XDR	‐
Kp 243	Internal medicine	Blood	Male	35	Non	4	8	XDR	‐
Kp 244	Infectious diseases	Urine	Female	41	Moderate	32	128	XDR	‐
Kp 245	Infectious diseases	Urine	Female	29	Strong	32	64	XDR	‐
Kp 246	Urology	Urine	Female	38	Non	2	8	XDR	‐
Kp 247	Men	Urine	Female	42	Strong	32	32	XDR	‐
Kp 248	Women	Blood	Female	50	Moderate	4	8	XDR	‐
Kp 249	Men	Blood	Male	48	Non	4	8	XDR	‐
Kp 250	Internal medicine	Urine	Female	38	Strong	8	16	XDR	‐
Kp 251	Men	Urine	Male	43	Strong	16	32	XDR	*ecpA*
Kp 252	Women	Blood	Female	42	Non	2	4	XDR	*ecpA*
Kp 253	Men	Sputum	Male	36	Moderate	4	32	XDR	*ecpA*
Kp 254	Pediatrics	Urine	Female	13	Moderate	8	16	XDR	*ecpA, ant(2”)‐Ia*
Kp 255	Pediatrics	Blood	Female	11	Moderate	4	8	XDR	*ecpA, aac(3′)‐IIa*
Kp 256	ICU	Sputum	Female	56	Strong	32	16	XDR	*ecpA*
Kp 257	ICU	Sputum	Male	74	Moderate	8	8	XDR	*ecpA*
Kp 258	ICU	Tracheal tube	Female	82	Non	4	8	XDR	*ecpA, FimA, ant(2”)‐Ia*
Kp 259	Surgery	Sputum	Female	53	Strong	64	32	XDR	*ecpA, FimA, FimH, ant(2”)‐Ia, aph(3′)‐Ia, aac(6′)‐Ib*
Kp 260	Men	Urine	Male	51	Non	8	8	XDR	*ecpA, FimA*
Kp 261	Men	Urine	Male	50	Strong	64	32	XDR	*ecpA*
Kp 262	Burn	Wound	Female	21	Strong	16	16	XDR	‐
Kp 263	Burn	Wound	Male	19	Moderate	8	32	‐	*ecpA, ant(2”)‐Ia*
Kp 264	ENT	Tonsil	Male	10	Non	4	8	‐	*ecpA, ant(2”)‐Ia*

Abbreviations: ICU, intensive care units; MBEC, minimal biofilm eradication concentration; MDR, multidrug resistant.

## 4 DISCUSSION

This study evaluated the aminoglycosides resistance and biofilm formation potency in *K. pneumoniae* strains collected from hospitalized patients in Ahvaz, southwest Iran. The isolates showed different resistance rates against aminoglycoside antibiotics with the highest and the lowest resistance to tobramycin and amikacin, respectively. In a previous study from China, the lowest aminoglycoside resistance was found to amikacin and streptomycin.^26^ Also, Nasiri et al.^27^ from Iran reported the lowest resistance rate to netilmicin and amikacin. Despite the fact that tobramycin is not available in the pharmaceutical markets of Iran, there was a high resistance to this antibiotic (71%) in this study, which was consistent with a previous study by Latifi et al.^28^ from Iran. This could be explained by the cross‐resistance phenomenon among aminoglycoside antibiotics. In a previous study by Jansen et al.^29^ from Germany, significant cross‐resistance among three aminoglycosides gentamicin, tobramycin, and streptomycin was observed in *Pseudomonas* isolates collected from cystic fibrosis patients.

Despite the emergence of resistant bacteria to various antibiotics, aminoglycosides are still one of the best options in the treatment of carbapenem‐resistant gram‐negative bacteria. However, the results of the current study did not confirm this fact. In this study, the majority of *K. pneumoniae* strains were resistant to at least one aminoglycoside, making them not proper treatment option especially in case of carbapenem‐resistant and MDR isolates. This observation was in contrast to a previous study by Galani et al.^30^ from Greece.

Indiscriminate and uncontrolled use of antibiotics, especially in third world countries like Iran, is one of the reasons for increasing antibiotic resistance. Two of the most principal mechanisms of resistance to aminoglycosides are AME and RMTase genes, which are known to be present in gram‐positive and gram‐negative bacteria. The existences of these coding genes has been reported in several studies from Iran.^28^, ^31^ In the current research, the backbone gene of RMTase, *armA*, was detected in 27% of the amikacin resistant strains that was higher than a previous report by Nasiri et al.^27^ (7.7%) from Iran. Also, the prevalence of *aac(3′)‐IIa* and *aac(6*′*)‐Ib* in our study was higher than that reported from previous studies from Iran and China in which 20.75%−30.2% and 19.8%−21.7% of clinical isolates of *K. pneumonia* were positive for the presence of *aac*(3)‐IIa and *aac*(6′)‐Ib genes, respectively.^28^, ^32^, ^33^ In our study *ant(2″)‐Ia* was the most frequent AME gene. In line with this study, Miller et al.^34^ reported the *ant(2″)‐Ia* gene as the dominant determinant in the United States, in which gentamicin was the most commonly used aminoglycoside. Furthermore, 75.7% of our isolates harbored combination of two or three AMEs and/or RMTase genes among which *armA‐ ant(2″)‐Ia, armA*‐*aac(6′)‐Ib*, and *aac(6′)‐Ib‐aac(3′)‐IIa* were more prevalent. The prevalence of combination of *armA* and AMEs genes reported by two studies from Greece^30^ and China^33^ was lower than this study. However, in contrast with our findings, in several studies the combination of *aac(6′)‐Ib and aac(3′)‐IIa* was reported as the most common genotype.^28^, ^34^

One of the notable findings in this study was the 100% rate of carbapenems resistance of *K. pneumoniae* isolates. Moreover, the majority of isolates were biofilm producers. These findings were in line with the previous studies that revealed significant correlation between biofilm formation capacity and antibiotic resistance among *K. pneumonia* isolates.^35^, ^36^, ^37^ Carbapenems are extremely successful against infections produced by MDR *K. pneumoniae* strains. MDR *K. pneumoniae* is a significant source of concern because it not only causes severe and fatal infections, but it also prolongs hospitalization, resulting in higher treatment costs.^36^

Our findings revealed that biofilm producer strains were significantly aminoglycosides non‐susceptible isolates that was in agreement with a previous study by Karimi et al.^38^ who showed biofilm‐producing *K. pneumoniae* isolates were more resistant to gentamicin and amikacin than those of non‐biofilm producers. In this study, the majority of XDR isolates were strong biofilm producers. In line with our results, Vuotto et al.^39^ showed a positive relationship between antibiotic resistance profile and biofilm‐forming ability in XDR *K. pneumoniae* strains. However, the presence of association between antibiotic resistance and biofilm formation capacity is still debated by some researchers.^40^, ^41^

This study showed that *ecpA, fimA, mrkD*, and *mrkA* genes were harbored by all biofilm producing *K. pneumoniae* strains, whereas *fimH* was determined in 93% (82/88) of isolates. The prevalence of *mrkA, ecpA*, and *fimH* (100%) in our study were almost similar to that reported by Alcántar‐Curiel et al.^21^ In addition, consistent with our results, Alcántar‐Curiel et al.,^21^ showed that 80% of *K. pneumoniae* isolates were able to produce biofilm and all isolates carried the *mrkA* gene. Moreover, our results showed that all MDR/XDR carbapenem‐resistant biofilm producing *K. pneumoniae* isolates carried the *ecpA, fimA, fimH, mrkD*, and *mrkA* genes. These results may indicate the importance of these virulence determinants in association with carbapenem resistance in *K. pneumoniae*. So far, no study has investigated the association of aforementioned genes with carbapenem resistance in *K. pneumoniae* isolates.

This study was performed during the pandemic of COVID‐19 and due to the crisis raised from this situation and the traffic restrictions, there were several limitations. In this study we did not evaluate the clonality of the isolates by genotyping methods. Also, we did not evaluate the other possible aminoglycoside resistance mechanisms including efflux pumps.

In conclusion, this study showed that all *K. pneumoniae* isolates were carbapenem‐resistant. Moreover, *K. pneumoniae* isolates showed the highest and the lowest aminoglycoside resistance rates to tobramycin and amikacin, respectively. Majority (more than 70%) of isolates were biofilm producers and there was significant association between antibiotic resistance pattern and the strength of biofilm production. Also, *ant(2”)‐Ia* and *aph(3′)‐Ia* were the most and the least frequent AME genes, respectively. Finally, most AMEs harboring isolates were also positive for presence of the biofilm related genes including *ecpA, mrkA*, and fimbrial genes.

## AUTHOR CONTRIBUTIONS

**Saeed Khoshnood**: Resources. **Sousan Akrami**: Conceptualization. **Morteza Saki**: Data curation. **Moloudsadat Motahar**: Formal analysis. **Sara Masihzadeh**: Resources. **Sara Daneshfar**: Resources. **Hossein Meghdadi**: Validation. **Effat Abbasi Montazeri**: Visualization. **Marjan Abdi**: Writing—original draft. **Zahra Farshadzadeh**: Conceptualization; investigation; supervision.

## CONFLICT OF INTEREST STATEMENT

The authors declare no conflict of interest.

## TRANSPARENCY STATEMENT

The lead author Zahra Farshadzadeh affirms that this manuscript is an honest, accurate, and transparent account of the study being reported; that no important aspects of the study have been omitted; and that any discrepancies from the study as planned (and, if relevant, registered) have been explained.

## ETHICS STATEMENT

Protocols for collection of samples as well as the experiment plan and all methods were performed in accordance with the guidelines and regulations of Ahvaz Jundishapur University of Medical Sciences and approved by the institutional ethical committees. Written informed consent was obtained from participants or parents before their involvement. The present research was supported by the Ahvaz Jundishapur University of Medical Sciences, Ahvaz, Iran (OG‐9838). All authors have provided consent for this publication.

## Data Availability

The authors confirm that the data supporting the findings of this study are available within the article. All data is in article.
