# Corrigendum to “**Molecular evaluation of aminoglycosides resistance and biofilm formation in** ***Klebsiella pneumoniae*** **clinical isolates: A cross‐sectional study**”

**DOI:** 10.1002/hsr2.1439

**Published:** 2023-07-24

Khoshnood S, Akrami S, Saki M, et al. Molecular evaluation of aminoglycosides resistance and biofilm formation in *Klebsiella pneumoniae* clinical isolates: a cross‐sectional study. *Health Sci Rep*. 2023;6:e1266. doi:10.1002/hsr2.1266

The Conclusion in the “Abstract” section was corrected as follows: “The *ant (2″)‐Ia*, *aac (3′)‐IIa* and *armA* genes were the most prevalent genes alone or in various combinations in aminoglycoside non‐susceptible isolates. Moreover, our findings present probable evidence for being amikacin as a useful aminoglycoside against biofilm producing carbapenem resistant *K. pneumoniae* isolates.”

The “Author Contributions” section for Saeed Khoshnood was corrected as follows: Saeed Khoshnood: Conceptualization; investigation.

We apologise for these errors.

